# Robust Detection of Out-of-Distribution Shifts in Chest X-ray Imaging

**DOI:** 10.1007/s10278-025-01559-7

**Published:** 2025-06-02

**Authors:** Fatemeh Karimi, Farzan Farnia, Kyongtae Tyler Bae

**Affiliations:** 1 Department of Diagnostic Radiology, University of Hong Kong, Hong Kong, China; 2 Department of Computer Science and Engineering, Chinese University of Hong Kong, Hong Kong, China

**Keywords:** Medical imaging, Chest X-ray, Out-of-distribution detection, Generative adversarial networks, MIMIC dataset, Optimization

## Abstract

This study addresses the critical challenge of detecting out-of-distribution (OOD) chest X-rays, where subtle view differences between lateral and frontal radiographs can lead to diagnostic errors. We develop a GAN-based framework that learns the inherent feature distribution of frontal views from the MIMIC-CXR dataset through latent space optimization and Kolmogorov–Smirnov statistical testing. Our approach generates similarity scores to reliably identify OOD cases, achieving exceptional performance with 100% precision, and 97.5% accuracy in detecting lateral views. The method demonstrates consistent reliability across operating conditions, maintaining accuracy above 92.5% and precision exceeding 93% under varying detection thresholds. These results provide both theoretical insights and practical solutions for OOD detection in medical imaging, demonstrating how GANs can establish feature representations for identifying distributional shifts. By significantly improving model reliability when encountering view-based anomalies, our framework enhances the clinical applicability of deep learning systems, ultimately contributing to improved diagnostic safety and patient outcomes.

## Introduction

The application of deep learning (DL) techniques in medical imaging analysis has grown significantly, offering promising solutions for tasks such as disease diagnosis and image classification. However, a critical challenge remains: the reliable detection of OOD inputs—samples that deviate significantly from the training data. In high-stakes environments like healthcare, where inaccurate predictions can lead to serious consequences, ensuring the dependability of machine learning models when encountering novel or unexpected data is paramount. Discrepancies between training data and real-world deployment data can adversely affect model performance, making OOD detection essential [1].

Classical deterministic algorithms, such as edge detection [2] and histogram-based methods [3], have been used to distinguish between lateral and frontal chest X-ray distributions. However, these methods often fail to handle complex variations and subtle distribution shifts. Our experiments with Canny edge detection, as well as a histogram-based method, revealed limitations. These methods achieved low accuracy, precision, recall, and F1 scores, underscoring their inability to reliably detect OOD samples. These findings highlight the need for more advanced approaches, such as GANs, which can model complex data distributions and capture subtle differences between image classes.

In this study, we assess the efficacy of GANs for OOD detection in medical imaging, focusing on chest X-rays. Our approach leverages GANs to predict outlying values in medical images by establishing an inherent feature distribution from a training dataset of frontal and lateral chest radiographs. When presented with a test sample, the model generates a similarity score to determine whether the sample is in-distribution (ID) or OOD. By iteratively optimizing a latent variable *z* using gradient-based methods, we monitor the Kolmogorov–Smirnov (K–S) test statistic and *p*-value to assess the goodness-of-fit between the generated sample and the target sample. If the *p*-value falls below 0.05, the sample is identified as OOD. This process enables the model to effectively distinguish between ID and OOD samples, even in the presence of subtle distribution shifts.

Through this study, we aim to highlight the limitations of current DL models in handling OOD inputs in medical imaging, propose a GAN-based framework for accurate OOD detection in chest X-ray analysis, and demonstrate the effectiveness of our approach in improving the accuracy and dependability of DL models in medical imaging applications. By addressing these challenges, we hope to contribute to the development of more reliable AI systems for healthcare, ultimately improving patient outcomes.

## Related Work

The calibration of modern neural network classifiers, particularly in terms of confidence estimates, often falls short, leading to incorrect predictions with high confidence and difficulty recognizing inputs that differ from the training data [4]. These models can fail silently, confidently making predictions even when presented with nonsensical inputs or adversarial attacks [5], raising significant concerns about AI safety [6]. This issue has prompted the development of methods for detecting OOD examples.

Several approaches have been proposed for OOD detection. Hendrycks et al. introduced a straightforward method that uses thresholding based on predicted softmax class probabilities [4]. Lee et al. improved upon this by proposing a solution involving a generator and classifier, where the generator creates OOD examples at the boundary of the data manifold, and the classifier assigns uniform class probabilities to these examples [7]. DeVries et al. introduced a technique in which classifiers output confidence estimates for each input, which helps differentiate between in- and out-of-distribution examples [8].

These methods have made significant progress in building more reliable classifiers. However, challenges remain, particularly with undetected out-of-distribution data points, which can pose risks in critical applications like healthcare [9]. To address the risks from dataset shifts, uncertainty quantification methods have been proposed [10]. These methods can be divided into two categories: model-specific approaches such as Bayesian neural networks and Monte Carlo dropout [11] and density estimators like variational autoencoders and normalizing flows [12], which aim to learn the distribution of the training data.

## X-ray OOD Detection Methods

In medical imaging, especially chest X-ray analysis, detecting inputs that deviate from the training distribution is crucial for ensuring model reliability. While various methods have been proposed, each has limitations that indicate the need for improved solutions.

The Maximum Softmax Probability (MSP) method, introduced by Hendrycks et al. Hendrycks, 2016 0.45Hendrycks, 2016 0.45Hendrycks, 2016 0.45Hendrycks, 2016 0.45Hendrycks, 2016 0.45 [4], classifies samples as OOD based on the highest softmax probability. However, MSP tends to overestimate confidence, leading to a high false positive rate, particularly in COVID-19 datasets, where it achieved an AUROC of only 52.85%. This limitation demonstrates that softmax-based confidence alone is insufficient for OOD detection in medical imaging.

ODIN enhanced MSP by adding temperature scaling and input perturbation [5]. However, it remains sensitive to preprocessing techniques and struggles to generalize in clinical settings, with a 46.69% AUROC on COVID-19 datasets. Mahalanobis distance–based methods classify test samples based on their distance from the training data distribution [13]. While effective in some settings, Mahalanobis distance assumes Gaussian distribution, which may not always hold in medical datasets, leading to unreliable results like an AUROC of 80.66% and a false positive rate of 68.69% on the IRMA dataset.

*K*-nearest neighbors (KNN)–based methods evaluate test samples by measuring their distance to the nearest neighbors [14]. While KNN showed promising results on COVID-19 datasets (80.50% AUROC), it is computationally expensive for large datasets, limiting its practical use in real-time clinical applications. Probabilistic distance methods leverage pairwise Mahalanobis distances to achieve performance but suffer from high computational complexity, making them unsuitable for real-time detection [15].

One-class classification methods are effective for detecting deviations from in-domain data as OOD [9]. However, they face challenges with multi-class datasets like chest X-rays, where pathologies vary significantly. Additionally, these methods rely on hyperparameter tuning, making them less adaptable to real-world clinical environments.

Recent work by Zamzmi et al. explored statistical process control (SPC)–based methods to monitor temporal shifts in medical imaging [16]. Although these methods can track deviations over time, they are not optimized for real-time OOD detection, highlighting the potential for combining SPC-based monitoring with generative models such as GANs for enhanced OOD detection.

The IDV method strengthens OOD detection consistency through majority voting across multiple model instances, achieving high performance on datasets like ChestX-ray [15, 17]. However, IDV requires extensive training data and may struggle to adapt to new distributions. Similarly, methods like FRODO (Free Rejection of Out-of-Distribution) utilize the Mahalanobis distance to measure how closely a test sample aligns with the training data distribution [18]. While effective, FRODO’s reliance on certain distributional assumptions may limit its applicability across diverse medical datasets. These limitations, such as overconfidence, reliance on distribution assumptions, computational inefficiency, and poor real-time adaptability, highlight the need for more advanced solutions for OOD detection.

Existing methods for OOD detection in medical imaging often overlook anatomical consistency, but recent advances in spine detection frameworks offer methodological parallels. Zhang et al. proposed U2 AD [19], an unsupervised approach for detecting T2 hyperintensities in spinal cord MRI. This approach demonstrates how uncertainty-guided masking can improve robustness to ambiguous anatomical variations. By leveraging vision transformers and Monte Carlo sampling, U2 AD highlights regions of uncertainty in spinal MRI, offering a paradigm for handling view-based shifts in chest X-rays.

Similarly, Skeleton-OOD [20], a graph-based method for skeleton data, encodes anatomical hierarchies to detect domain shifts. It applies activation shaping (ASH) to filter noise and amplify discriminative features, retaining in-distribution classification capability while improving OOD sensitivity.

While these methods focus on spinal structures in MRI or skeleton data, their core innovations, namely uncertainty-aware feature prioritization, anatomical graph modeling, and iterative optimization, offer transferable solutions to the challenges of chest X-ray OOD detection. Our GAN-based framework diverges by directly learning feature distributions through adversarial training and statistical validation (K–S testing), avoiding dependencies on pre-defined anatomical hierarchies.

## GAN-Based OOD Detection Methods

Recent advancements in GANs have enhanced OOD detection. Dionelis et al. proposed TailGAN, which generates boundary samples by minimizing a multi-term loss function to target low-probability regions of ID data [21]. While TailGAN achieves competitive AUROC scores on datasets like CIFAR-10, its clinical utility faces limitations: reliance on base GAN accuracy, high computational costs from dual training stages, and poor generalization to unseen anomalies.

Marek et al. introduced OodGAN, a method that simplifies OOD text generation using SeqGAN to synthesize OOD utterances directly via adversarial rewards [22]. Though effective for dialog systems, its scalability suffers from training instability, post-processing overhead, and dependence on high-quality in-domain data.

Azizmalayeri et al. developed the adversarially trained discriminator (ATD), which integrates adversarial training with generative modeling [23]. This approach leverages pre-trained feature extractors and perturbed ID/OOD data to resist attacks However, fixed OOD datasets and computational demands limit its adaptability to novel anomalies.

In medical imaging, StyleGAN2-based methods focus on detecting OOD samples in liver CTs through reconstruction errors [24]. Their clinical adoption remains constrained by computational intensity, transfer learning dependencies, and unvalidated generalization to new artifacts.

Kwon et al. proposed PGGAN, which employs progressive GANs for high-resolution anomaly detection [25]. Segmentation prerequisites and manual threshold tuning hinder scalability. Conversely, AdverX-Ray detects hardware artifacts in 128 × 128 patches using a lightweight adversarial VAE but overlooks global anomalies and struggles with unseen devices [26].

These methods highlight critical gaps, computational inefficiency, contextual blindness, and insufficient validation for clinical settings. Our work addresses these challenges with a streamlined GAN framework. It eliminates segmentation dependencies, integrates statistical validation via the Kolmogorov–Smirnov (K–S) test, and detects holistic distribution shifts in full-resolution chest X-rays.

## Kolmogorov–Smirnov Test for OOD Detection

The Kolmogorov–Smirnov (K–S) test is a non-parametric statistical method that quantifies the divergence between two distributions by measuring the maximum difference in their empirical cumulative distribution functions [27]. Its distribution-free nature makes it particularly valuable for scenarios where the underlying data distribution is unknown. For instance, Cheng et al. [28] leveraged the Kolmogorov–Smirnov (K–S) test for change point detection in time-series data, integrating it with a matched filtering mechanism to suppress false positives while retaining sensitivity to statistically significant shifts. This work underscores the K–S test’s effectiveness in identifying distributional discrepancies without parametric assumptions, a property that aligns well with OOD detection challenges.

Building on this foundation, Jiang et al. [29] proposed a group-wise OOD detection framework that combines flow-based models with the K–S test. By mapping input data to a latent space via invertible transformations, they applied the K–S test to compare test samples against training data or predefined priors. While effective in their tested domains, their method’s reliance on invertible architectures and random projections introduces practical constraints. Specifically, the requirement for invertibility limits flexibility in model design, and the latent-space comparisons obscure spatial interpretability, a critical shortcoming in medical imaging, where pixel-level reliability and anatomical coherence directly impact diagnostic utility.

To address these limitations, we adapt the K–S test for medical imaging by integrating it into a GAN framework. Unlike Jiang et al.’s latent-space approach, our method computes similarity scores directly in the sample space of chest X-rays, bypassing the need for restrictive invertible transformations.

## Method

### Data

In this research, we utilize the MIMIC-CXR dataset [30], which is a comprehensive collection of chest X-ray images. The dataset contains over 370,000 images from more than 63,000 patients. For our study, we train a GAN using frontal chest radiographs as the ID data, while lateral chest radiographs are treated as OOD data. Additionally, we also explore scenarios where lateral radiographs are considered ID data and frontal radiographs serve as OOD data.

### GAN Training for MIMIC Frontal Chest Radiographs

**Fig. 1:**
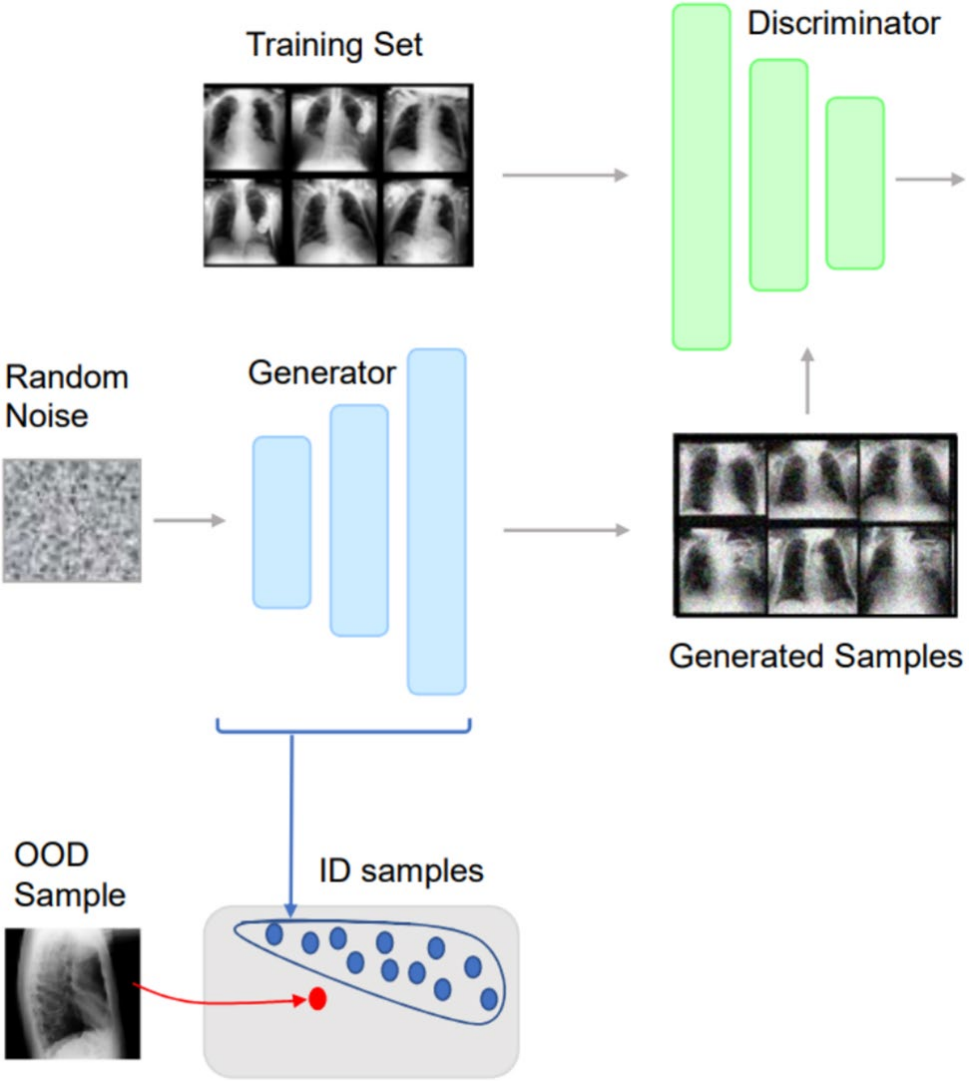
Schematic description of our method. Using GANs trained on frontal images from the MIMIC dataset, the generator learned the data distribution

In our research, we use GANs to train our models on frontal and lateral chest radiographs obtained from the MIMIC dataset (Fig. 1). GANs consist of two neural networks: a generator and a discriminator. The generator creates synthetic data that mimics the distribution of the real data, while the discriminator evaluates whether the data it receives is real (from the dataset) or generated (from the generator).

**Fig. 2:**
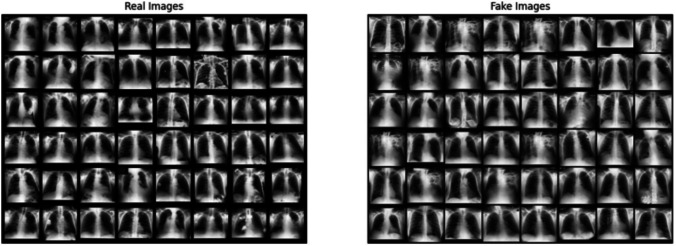
Real and fake frontal chest radiographs. The real frontal radiographs served as the training dataset for the discriminator, while the fake radiographs were generated by the generator. Through competition during training, the generator learned to create images resembling realistic data

The training process is framed as a zero-sum game, where the generator aims to produce data indistinguishable from real data, and the discriminator aims to correctly identify whether the data is real or generated (Fig. 2). This adversarial process continues until the generator produces highly realistic data that the discriminator can no longer reliably distinguish from real data. This setup allows the GAN to effectively learn the underlying distribution of the dataset.

Specifically, we develop two separate models: The first model is trained on frontal chest radiographs as the ID data and lateral chest radiographs as the OOD data. Similarly, the second model is trained on lateral chest radiographs as the ID data and frontal chest radiographs as the OOD data. The GAN architecture comprises two primary components: the generator and the discriminator. We use a custom weight initialization function to set the initial weights for both networks[31].

The generator network is structured with multiple ConvTranspose2 d [32], BatchNorm2 d [33], and ReLU activation layers [34], culminating in a Tanh activation function in the final output layer [35]. The discriminator network includes Conv2 d [36], BatchNorm2 d, and LeakyReLU activation layers [37] and a Sigmoid activation function in its output layer [38].

We apply the binary cross entropy loss function and the Adam optimizer [12], with a learning rate of 0.001 and beta values of (0.5, 0.999), to train both networks. The GAN undergoes 999 training steps to achieve optimal performance.

Throughout the training process, we generate a batch of latent vectors *z* to monitor the generator’s progression. The generator’s objective is to create realistic images that capture the distribution of frontal chest radiographs, making it challenging for the discriminator to distinguish them from actual MIMIC frontal images. This method effectively models the distribution of latent variables, enabling the identification of OOD samples in medical imaging analysis.

### Optimization problem

In GANs, the generator function G takes a latent variable z as input and produces a generated sample G(z). The goal is to minimize the discrepancy between G(z) and a target sample X by optimizing z. This is achieved by minimizing the Euclidean distance between *G*(*z*) and X using gradient-based optimization techniques, such as the Adam optimizer with a learning rate of 0.001.

To detect OOD samples, we used the K–S test to compare the distribution of generated samples with the target distribution. If the *p*-value from the K–S test was below the significance level (0.05), the sample was identified as OOD.

In our study, we tested for distribution shifts between lateral and frontal chest X-ray images. For detecting lateral distribution from frontal, we used a specific loss threshold and optimization steps. Once the loss reached the threshold, we performed the K–S test. If the *p*-value was above 0.05, the sample was considered ID; otherwise, it was flagged as OOD. A similar process was applied for detecting frontal distribution from lateral images.

By iteratively optimizing *z* and monitoring the K–S test results, we effectively distinguished between ID and OOD samples, enabling accurate detection of distribution shifts in medical imaging.
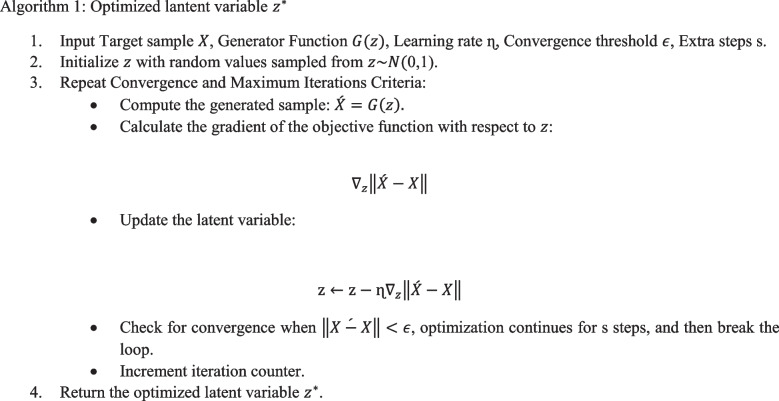


## Result

### Performance Evaluation of the GAN-Based Approach

**Fig. 3:**
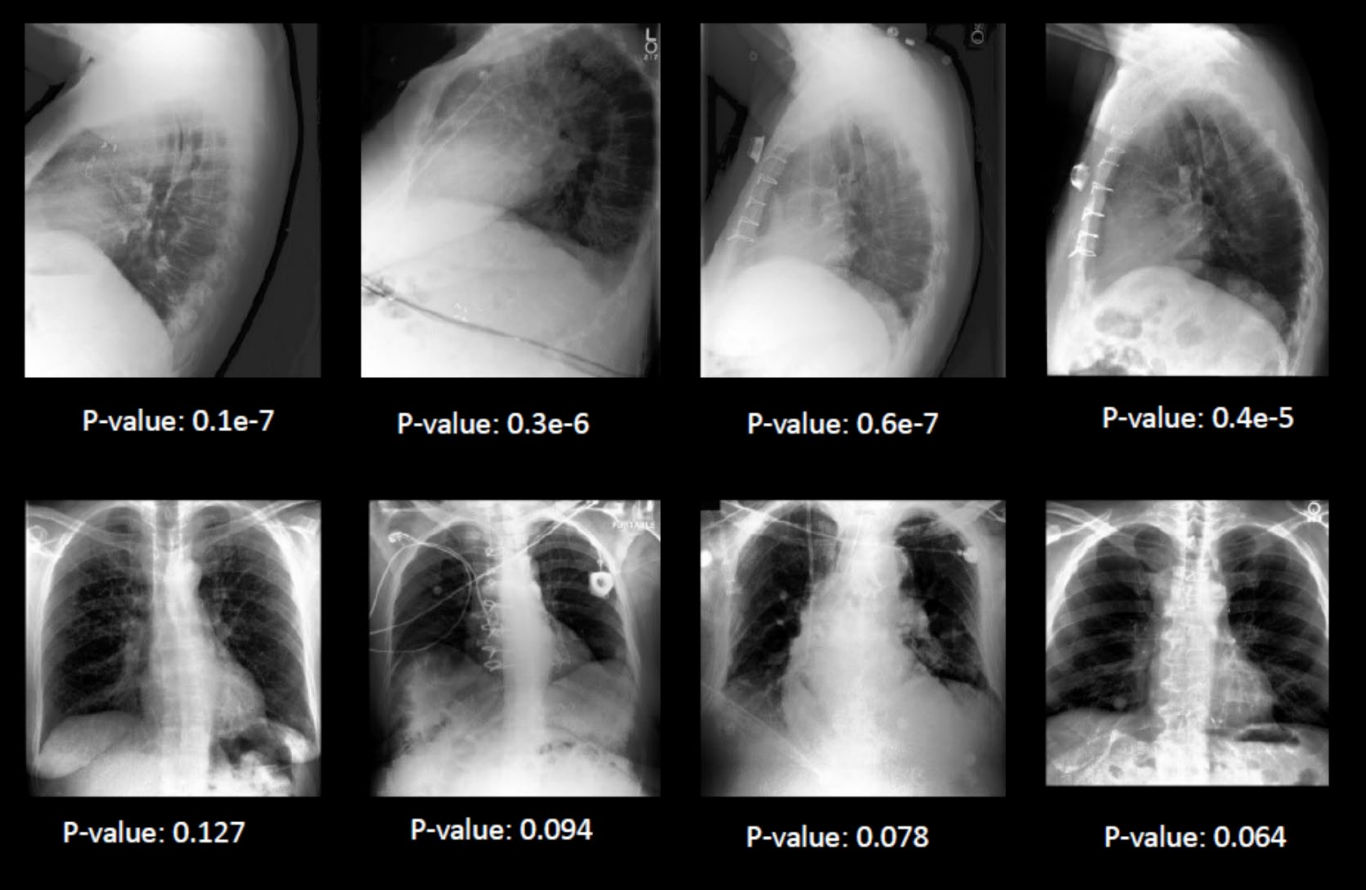
Several examples of frontal and lateral chest radiographs with successful discrimination performance (as denoted by the *p*-values)

**Fig. 4:**
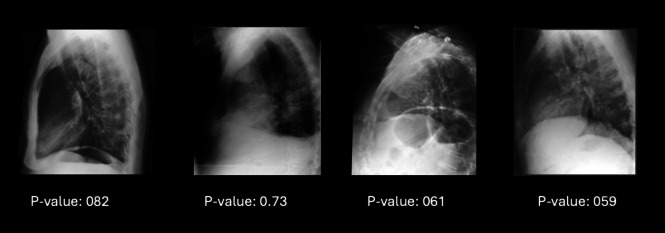
Example of lateral chest radiographs that failed in discrimination (as denoted by the *p*-values)

We evaluated the performance of our GAN-based method for detecting OOD samples in chest X-ray images using the MIMIC dataset. The results, summarized in Tables 1 and 2, demonstrate that our approach successfully distinguished OOD cases with high accuracy, precision, recall, and F1 score. Figure 3 shows several examples of frontal and lateral chest radiographs with successful discrimination performance, whereas Fig. 4 presents a few lateral chest radiograph cases that failed in discrimination.

**Table 1:** Detection of lateral distribution from frontal using GAN-based approach

Hyperparameters	Accuracy	Precision	Recall	F1 score
Loss-threshold = 0.2 Extra-step = 100	0.825	0.83	0.821	0.825
Loss-threshold = 0.19 Extra-step = 100	0.875	0.88	0.872	0.875
Loss-threshold = 0.18 Extra-step = 100	0.925	0.93	0.92	0.925
Loss-threshold = 0.17 Extra-step = 100	0.94	0.95	0.94	0.94
Loss-threshold = 0.15 Extra-step = 95	*0.975*	*1.0*	*0.95*	*0.974*

Italicized values indicate the highest performance metrics

**Table 2:** Detection of frontal distribution from lateral using GAN-based approach

Hyperparameters	Accuracy	Precision	Recall	F1 score
Loss-threshold = 0.2 Extra-steps = 60	0.78	0.79	0.77	0.78
Loss-threshold = 0.25 Extra-steps = 60	0.80	0.79	0.814	0.802
Loss-threshold = 0.3 Extra-steps = 60	0.825	0.83	0.821	0.825
Loss-threshold = 0.35 Extra-steps = 60	0.875	0.88	0.872	0.875
Loss-threshold = 0.4 Extra-steps = 60	*0.925*	*0.93*	*0.92*	*0.925*

Italicized values indicate the highest performance metrics

For detecting lateral images as OOD, the best results were achieved with a loss threshold of 0.15, yielding an accuracy of 97.5%, precision of 1.0, recall of 0.95, and an F1 score of 0.974 (Table 1).

Similarly, for detecting frontal images as OOD, the best results were obtained with a loss threshold of 0.4, where the model achieved an accuracy of 92.5%, precision of 0.93, recall of 0.92, and an F1 score of 0.925 (Table 2). These findings highlight the effectiveness of our GAN-based approach in accurately identifying OOD samples.

### Hyperparameter Analysis

To investigate the impact of hyperparameters on model performance, we conducted an analysis of variance (ANOVA) on the accuracy results presented in Tables 1 and 2. The analysis revealed statistically significant differences in accuracy across different hyperparameter settings for both lateral and frontal distribution detection.

For lateral image detection, ANOVA results indicated a highly significant effect of the loss threshold (*F*(4,10) = 271.875, *p* < 0.001). Similarly, for frontal image detection, ANOVA confirmed significant differences in accuracy (*F*(4,10) = 197.386, *p* < 0.001), with a loss threshold of 0.4 yielding the best results.

### Comparison with Classical Methods

To evaluate the effectiveness of our GAN-based approach, we compared it with classical deterministic algorithms, including Canny edge detection [2] and a histogram-based method [3]. The results are presented in Table 3.

**Table 3:** Detection of lateral distribution from frontal

Method	Accuracy	Precision	Recall	F1 score
Canny edge detection [2]	0.83	0.78	0.91	0.84
Histogram-based method [3]	0.735	0.725	0.755	0.740
Our GAN-based approach	*0.975*	*1.0*	*0.95*	*0.974*

Italicized values indicate the highest performance metrics

For Canny edge detection [2], threshold selection was performed on frontal (ID) data. We conducted a grid search over plausible threshold values to maximize the F1 score. The method achieved an accuracy of 83% and an F1 score of 84%, with precision and recall scores of 0.78 and 0.91, respectively. For the histogram-based method [3], we performed a grid search over thresholds to maximize the F1 score. The optimal threshold achieved an accuracy of 73.5%, precision of 0.725, recall of 0.755, and F1 score of 0.740.

## Discussion

Our study demonstrates the effectiveness of a GAN-based approach for OOD detection in chest X-ray imaging. It addresses key challenges that have not been fully explored in the literature. While previous research has mainly focused on natural image datasets or relied on softmax confidence scores and Mahalanobis distances, these methods often struggle with medical imaging due to the complexity of clinical data [4, 13]. Many existing OOD detection techniques, such as ODIN, Mahalanobis distance-based methods, and *K*-nearest neighbors (KNN) classifiers, rely on predefined statistical assumptions and are highly sensitive to dataset shifts [5, 15]

Our GAN-based method takes a more adaptive and data-driven approach. Instead of depending on fixed statistical assumptions, it learns the underlying distribution of ID samples and detects deviations from this learned feature space [29]. Unlike traditional softmax-based confidence scores, which assume a Gaussian distribution, our method uses the Kolmogorov–Smirnov (K–S) test to statistically validate deviations. This provides a more reliable and interpretable framework for OOD detection. Our results show that this approach significantly outperforms classical edge detection and histogram-based methods [2, 3], achieving 100% precision and 97.5% accuracy in detecting lateral chest X-rays as OOD. Additionally, our analysis of hyperparameters highlights the role of loss thresholds in improving detection performance, emphasizing the need for fine-tuning in clinical applications.

While GANs are commonly used for data augmentation and synthetic image generation, their potential for OOD detection in medical imaging remains underexplored [25, 26]. Our study contributes to this field by showing that GANs can effectively model the distribution of medical images and identify subtle shifts. This advancement is particularly useful in clinical settings, where detecting and excluding OOD samples improves diagnostic confidence and model reliability.

Despite its strong performance, our approach has limitations. It focuses on detecting OOD cases based on variations in the view of the same organ, specifically distinguishing frontal from lateral chest X-rays. This is more challenging than identifying OOD samples from different organs, as the differences are often subtle. Additionally, our study is limited to adult chest X-rays. Future research could explore its applicability to pediatric imaging, where anatomical structures and disease patterns differ significantly. Expanding the method to both pediatric and adult populations would provide deeper insights into its generalizability and clinical value.

Overall, our findings highlight the potential of GAN-based models for improving OOD detection in medical imaging. By addressing the weaknesses of traditional methods and introducing a statistically grounded framework, our study offers a new way to enhance the reliability of deep learning models in clinical practice. Further research could expand this approach to other imaging modalities and patient populations, contributing to more robust and trustworthy AI-driven healthcare solutions.

## Conclusion

In conclusion, we presented a GAN-based approach to improve the accuracy and reliability of deep learning models in detecting OOD samples in chest X-ray images. Our method successfully distinguished between frontal and lateral chest X-ray images, achieving a 100% detection performance for OOD cases. In a clinical diagnostic setting, our solution may be used to detect and exclude lateral chest X-ray images that can disturb the performance of AI programs, which are trained solely to process frontal chest X-ray images. Further research is needed to explore the generalizability of this approach to other medical imaging modalities and optimize the choice of hyperparameters for improved OOD detection performance.

## Data Availability

The dataset used in this study is publicly accessible and can be downloaded from https://physionet.org/content/mimic-cxr/2.1.0/.

## Code Availability

The code used in this study can be obtained from the first author upon request.
